# Innovative Treatments to Counteract Endothelial Dysfunction in Chronic Kidney Disease Patients

**DOI:** 10.3390/biomedicines12051085

**Published:** 2024-05-14

**Authors:** Giulia Marrone, Kevin Cornali, Manuela Di Lauro, Maria Josè Ceravolo, Luca Di Marco, Simone Manca di Villahermosa, Anna Paola Mitterhofer, Annalisa Noce

**Affiliations:** 1 Department of Systems Medicine, University of Rome Tor Vergata, 00133 Rome, Italycornali.kevin@hotmail.it (K.C.); luca.dimarco_1969@libero.it (L.D.M.); mncsmn00@med.uniroma2.it (S.M.d.V.); annapaola.mitter@uniroma2.it (A.P.M.); 2 Nephrology and Dialysis Unit, Department of Systems Medicine, University Hospital of Rome Tor Vergata, 00133 Rome, Italy

**Keywords:** endothelium, endothelial dysfunction, chronic kidney disease, nitric oxide, inflammation, oxidative stress, insulin resistance, bioactive natural compounds, ketoanalogues, innovative treatments

## Abstract

In chronic kidney disease (CKD) patients, several risk factors contribute to the development of endothelial dysfunction (ED), which can be described as an alteration in the cell structure or in the function of the endothelium. Among the well-known CKD-related risk factors capable of altering the production of endothelium-derived relaxing factors, we include asymmetric dimethylarginine increase, reduced dimethylarginine dimethylamine hydrolase enzyme activity, low-grade chronic systemic inflammation, hyperhomocysteinemia, oxidative stress, insulin resistance, alteration of calcium phosphorus metabolism, and early aging. In this review, we also examined the most important techniques useful for studying ED in humans, which are divided into indirect and direct methods. The direct study of coronary endothelial function is considered the gold standard technique to evaluate if ED is present. In addition to the discussion of the main pharmacological treatments useful to counteract ED in CKD patients (namely sodium–glucose cotransporter 2 inhibitors and mineralocorticoid receptor antagonist), we elucidate innovative non-pharmacological treatments that are successful in accompanying the pharmacological ones. Among them, the most important are the consumption of extra virgin olive oil with high intake of minor polar compounds, adherence to a plant-dominant, low-protein diet (LPD), an adaptive physical activity program and, finally, ketoanalogue administration in combination with the LPD or the very low-protein diet.

## 1. Introduction

In physiological conditions, an intact endothelium constitutes a barrier between blood circulation and the vascular wall. It acts as a modulator of blood circulation itself, and it is responsible for the regulation of vascular tone [1]. The endothelium regulates the production of a series of endothelium-derived relaxing factors, which are responsible for the maintenance of vascular homeostasis [2].

An intact endothelium exerts various functions. (i) An anti-inflammatory action, inhibiting the monocytes’ adhesion. In fact, the healthy endothelium prevents the adhesion of circulating monocytes at the level of the intimal through the production of different adhesion molecules, i.e., vascular cellular adhesion molecule-1 (VCAM-1). The latter plays a crucial role in the formation of atheromatous plaque [3]. (ii) An antithrombotic action through the release of nitric oxide (NO), a powerful vasodilator and antithrombotic agent that prevents platelets’ migration and aggregation [4]. (iii) An anticoagulant and profibrinolytic action. (iv) An anti-proliferative action, with the inhibition of the smooth muscle cells’ proliferation and their migration [5].

Endothelial dysfunction (ED) can be described as an alteration in the cell structure or in the function of the endothelial tissue that internally lines blood vessels [6]. ED plays a key role in the etiopathogenesis of numerous pathologies, so its identification and its treatment, using both traditional and innovative therapies, becomes of particular importance for the clinical management of patients affected by chronic degenerative non-communicable diseases, such as cardiovascular (CV) diseases, arterial hypertension, diabetes mellitus, and chronic kidney disease (CKD) [7,8].

CKD causes ED through several mechanisms that include the reduction of NO, the stimulation of the inflammatory response, and the increase of oxidative stress (OS), vascular permeability, and leukocyte adhesion, resulting in an angiogenesis impairment [5]. This review’s aims are to clarify the main ED risk factors in CKD patients, to describe its most innovative diagnostic methods, and, finally, to discuss its main pharmacological and non-pharmacological treatments, both traditional and innovative.

## 2. Search Methods

A literature search was conducted up to March 2024 according to the basic literature online search process. Articles from online databases belonging to PubMed, Scopus, and Cochrane Library were manually retrieved. For the paper search, we used the keyword “endothelial dysfunction” either alone or in combination with “asymmetric dimethylarginine” AND “eNOS” AND “inflammatory status” AND “hyperhomocysteinemia” AND “oxidative stress” AND “insulin resistance” AND “calcium-phosphorus metabolism” AND “early aging” AND “diagnosis of endothelial dysfunction” AND “therapeutic approaches” AND “drug therapies” AND “nutritional therapies”. The search included only papers in the English language with abstracts and reviews, original articles, and meta-analyses.

## 3. The Risk Factors of Endothelial Dysfunction in CKD Patients

**Figure 1:**
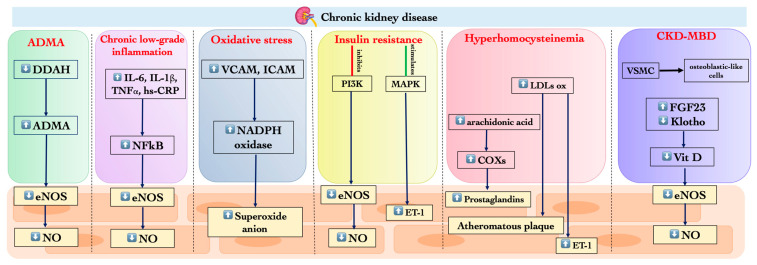
Factors involved in ED genesis in CKD. Abbreviations: ADMA, asymmetric dimethylarginine; CKD, chronic kidney disease; COX, cicloxigenase; DDAH, dimethylarginine imethylaminohydrolase; ED, endothelial dysfunction; eNOS, endotelial nitric oxide synthase; ET-1, endothelin-1; FGF23, fibroblast growth factor 23; hs-CRP, high-sensitivity C-reactive protein; ICAM, intercellular adhesion molecule; IL, inteleukin; LDL, low-density lipoprotein; MAPK, mitogen-activated protein kinase; NF-kB, nuclear factor kappa-light-chain-enhancer of activated B cells; NO, nitric oxide; PI3K, phosphoinositide 3-kinases; TNF, tumor necrosis factor; VCAM, vascular cell adhesion molecule; VSMC, vascular smooth muscle cells; ↑ increase; ↓ decrease.

ED is a common comorbidity of CKD, and it increases the risk for CV diseases. In this section, we examine the role of asymmetric dimethylarginine (ADMA), low-grade chronic systemic inflammation, hyperhomocysteinemia (HHcy), OS, insulin resistance (IR), alteration of calcium phosphorus metabolism, and early aging in ED onset and progression (Figure 1).

### 3.1. Asymmetric Dimethylarginine

NO production occurs by means of three different enzymes: neuronal nitric oxide synthase (nNOS), inducible NOS (iNOS), and endothelial NOS (eNOS). The latter is localized on the plasma membrane invaginations at the level of the endothelial cells of the blood vessels where, in physiological conditions, it produces NO at low concentrations [9]. In addition to promoting endothelium vasodilation, NO is involved in the correct functioning of the endothelium itself; in fact, it reduces the platelets’ aggregation, the migration and the proliferation of smooth muscle cells, the adhesion of monocytes, the expression of adhesion molecules, and the oxidation of low-density lipoprotein (LDL) cholesterol [10].

In this context, a strong endogenous inhibitor of eNOS is the ADMA, i.e., a dimethylated amino acid that is produced from L-arginine in endothelial cells. In CKD patients, the lower NO production is due to not only the high ADMA levels but also the reduced activity of dimethylarginine dimethiamine hydrolase (DDAH), the enzyme responsible for ADMA degradation [11].

ADMA is metabolized by the kidney and excreted in the urine; for this reason, in conditions of impaired renal function, its excretion is reduced. Furthermore, the alteration of the renal parenchyma leads to a decrease in the concentration of DDAH, resulting in ADMA accumulation [12]. Being related to a lower NO availability, ADMA is involved in the uncoupling of eNOS, which is responsible for free radical production [5].

### 3.2. Low-Grade Chronic Systemic Inflammation

Low-grade chronic systemic inflammation related to CKD is in part due to the activation of the innate immune system cells, including monocytes, macrophages, and granulocytes, and it is a frequent cause of ED [13,14].

In CKD patients, inflammation is present not only systemically but also locally in the kidneys. In fact, in the case of renal dysfunction, chronic low-grade inflammation triggers resident kidney cells to produce proinflammatory cytokines and chemokines and induces the deposition of the extracellular matrix (ECM), thus contributing to tubulointerstitial fibrosis [15]. As such, chronic low-grade inflammation is an important driver of CKD progression [14].

In nephropathic patients, an imbalance was observed between the production of the anti-inflammatory and proinflammatory cytokines to the advantage of the latter, with a serum increase of interleukin (IL)-1β, IL-6, tumor necrosis factor-α (TNF-α), and high-sensitivity C-reactive protein (hs-CRP) due to either their augmented release or their reduced kidney clearance [16].

Low-grade chronic systemic inflammation, in association with OS and the production of the advanced glycation end-products (AGEs), leads to the activation of the nuclear factor kappa B (NF-kB) pathway, resulting in lower eNOS enzyme activity and, consequently, lower NO bioavailability. These data are corroborated by an in vivo study conducted on CKD patients under conservative therapy. The authors evaluated, in 64 CKD patients, the arterial stiffness and vascular endothelial function compared to those of healthy subjects (the control group). Therefore, the authors demonstrated that the CKD patients exhibited a greater arterial stiffness and a lower vascular endothelial function compared to the control group, highlighting the role of OS and inflammation in ED observed in CKD patients under conservative therapy [17].

In this regard, CKD induces a constant and progressive activation of the endothelium with the release of soluble adhesion molecules (like the intracellular adhesion molecule ICAM-1, VCAM-1, and the von Willebrand factor (vWF)). These factors are capable of activating the NK-kB pathway, with possible damage at the level of both the endothelial and matrix cells [18]. Moreover, inflammation in several pathological conditions seems to reduce the serum levels of triiodothyronine [19,20]. Currently, although the relationship between inflammation and lower triiodothyronine levels is not completely clarified, it can be hypothesized that it is able to mediate ADMA’s negative impact on the endothelium [5,21].

In CKD, inflammation has been recognized since the late 1990s, when it was linked to CV diseases, protein–energy wasting (PEW) syndrome, and mortality [22]. Persistent inflammation in CKD is not only related to CV outcomes, including early atherosclerosis; it is also one of the key players in the development of PEW syndrome [23].

Finally, the low-grade inflammatory state is also related to the gut dysbiosis that characterizes CKD patients [24]. In fact, in those patients, proteolytic fermentation is increased compared to the saccharolytic ones. This implies an enhanced release of gut-derived uremic toxins, such as p-cresyl sulfate (pCS), trimethylamine n-oxide (TMAO), and indoxyl sulfate (IS), and a reduction in the production of short-chain fatty acids (SCFAs), such as acetate, propionate, and butyrate, compounds that exert healthy effects [25,26]. The composition of the gut microbiota in CKD patients is completely different from that of healthy subjects [27]. Moreover, dysbiosis induced by uremia is attributable to several factors and, with the decline in renal function, the colon assumes the role of the excretory organ [26]. In CKD, an increase was observed in the colon’s pH, which induces a selection of urease-positive species responsible for the conversion of urea into ammonia. This leads to a degradation of the mucus, which physiologically acts as a protective layer, and consequently alters the intestinal permeability due to the destruction of tight junctions [28].

Dysbiosis is also worsened by traditional nutritional management of the nephrotic patient, especially in the more advanced stages, which provides for a strict restriction of fibers and further unbalances microbial metabolism in the direction of proteolytic fermentation [29]. Therefore, in CKD, the gut microbiota must be considered a new CV risk factor, which can be modified through the inclusion of fibers in the diet or by following a Mediterranean diet (MD) [30,31,32].

Among the various dietary supplementations suggested to counteract the inflammation in CKD, there are omega-3 fatty acids, catechins, pomegranate, soy isoflavones, fibers, and probiotics [33,34,35].

Although further studies are needed to better clarify the molecular relationship between metabolites derived from the intestinal microbiota and CKD progression [36], all literature results indicate an involvement of gut dysbiosis in the onset of kidney disease and its progression, thus providing interesting perspectives for those therapeutic interventions aimed at modulating the gut–kidney axis [37].

### 3.3. Hyperhomocysteinemia

Homocysteine (Hcy) is a sulfur-containing amino acid that is generated by the demethylation of methionine. Hcy’s physiological plasma concentration is between 5 and 15 μmol/L, while values above 15 μmol/L indicate a condition of HHcy. The latter is induced by different factors, and the main ones include genetic and acquired ones. Among genetic factors, the most studied is the genetic polymorphism of methylenetetrahydrofolate reductase (MTHFR), while among acquired factors, there are gender, age, lifestyle, and CKD factors [12]. In fact, HHcy is observed in approximately 85% of CKD patients, due to both an altered metabolism of Hcy and its reduced excretion by the kidneys, which does not allow Hcy to be eliminated in the form of cysteine [38].

The HHcy condition is directly capable of provoking ED through several mechanisms. In fact, Hcy is enzymatically converted in Hcy thiolactone, which is able to thiolate the free amino groups of LDLs, thus forming oxidized LDLs. This condition results in macrophage aggregation and their adhesion to the endothelium. The homocysteinylated LDLs release Hyc thiolactone within the vascular wall, which leads to the phenomenon of intimal injury, the oxidation of cholesterol and unsaturated lipids, the platelets’ aggregation, myointimal hyperplasia, the deposition of sulfated glycosaminoglycans, fibrosis, and the calcification of atherosclerotic plaques [39].

On the other hand, HHcy also appears to be indirectly involved in ED through various mechanisms.

(i). The reduction of the NO bioavailability. In fact, at high Hcy concentrations, NO seems to react with the Hcy thiol group, thus reducing its bioavailability [40].
(ii). The increase in the production of prostaglandins and thromboxanes via the arachidonic acid–prostanoids pathway. In fact, Hcy seems to increase the release of arachidonic acid and its conversion into inflammatory molecules through cyclooxygenase (COX) enzymes [41].
(iii). The activation of the angiotensin II type 1 (AT1) receptor. It has been highlighted how HHcy is capable of activating the AT1 receptor signaling pathway, thus provoking a vasocontractile response through the release of prostanoids [42].
(iv). The increased production of reactive oxygen species (ROS), which, consequently, induces OS [43]. In fact, the abnormal production of ROS, provoked by HHcy, is able to damage the endothelial cells of the arterial wall and cause the modification of intracellular endothelial redox homeostasis. Moreover, OS is capable of inducing mitochondrial dysfunction and eNOS uncoupling, resulting in a decrease in the NO’s bioavailability and in the worsening of ED [44,45].
(v). The activation of endothelin-1 (ET-1), an important biomarker of ED [42]. The ET-1 is a powerful endogenous vasoconstrictor that is released by endothelial cells, and it can cause vascular cell fibrosis and can increase the release of ROS and proinflammatory cytokines [46].

However, in dialysis patients, the phenomenon of “reverse epidemiology” was described, as the conventional CV risk factors that are directly related to an increased risk of mortality in the general population paradoxically seem to be protective in hemodialysis patients. In particular, Kalantar-Zadeh described an inverse association between total Hcy plasma levels and the risk of mortality in end-stage renal disease (ESRD) patients. It is very important to consider that ESRD patients with and without CV history had Hcy plasma levels higher than those of the general population [47]. Moreover, another study analyzed the possible “reverse epidemiology” of total Hcy plasma and mortality in ESRD patients, demonstrating that after adjusting for confounding factors (namely, the inflammation and nutritional biomarkers), in this patient population, higher Hcy levels were also related to increased CV risk [48].

### 3.4. Oxidative Stress

OS is defined as an imbalance between the production of ROS and the ability to neutralize them through an antioxidant defense system [49]. In CKD patients, Ang II, shear stress, and hyperglycemia aggravate ROS production via NADPH oxidase, which affects cell metabolism, and it can also trigger severe cell damage until developing ED [50]. Moreover, the augmented OS in the setting of the uremic milieu via disruption of NO pathways promotes the enzymatic modification of circulating lipoproteins and lipids, the proteins’ carbamylation, and ED itself [51].

ROS production is greatly increased in CKD patients, partially because of an altered activation of the nuclear factor derived from erythroid 2 (Nrf2), which results in the downregulation of antioxidant and cytoprotective molecules [52]. Several endothelial cell enzymes, including xanthine oxidase, NADPH oxidase, and eNOS itself in its uncoupled form, can produce superoxide anions. In particular, the phenomenon of eNOS uncoupling has been observed in conditions of tetrahydrobiopterin (BH4) deficiency [53].

Physiologically, NO is produced by endothelial cells under the acetylcholine effect (after parasympathetic stimulation) or under shear stress exerted on the arterial walls [54]. Factors involved in the development of a pro-oxidant state, as occurs in CKD, should be both endogenous factors (such as mitochondrial dysfunction and NADPH oxidase overactivation) and exogenous ones (namely, cigarette smoke, pollution, certain drugs, radiation, and specific foods). They can contribute to decreased NO levels [55]. Among other factors that can influence NO biosynthesis, the transport of L-arginine in endothelial cells and the shifting of this amino acid in other pathways, such as those involving arginase, can contribute to its reduced levels [56]. Furthermore, ROS decreases NO’s bioavailability and promotes the generation of peroxynitrite, which causes DNA, proteins, lipids, and carbohydrates oxidative damage [57].

The monitoring of OS biomarkers, such as the evaluation of total antioxidant capacity in the blood (like the free oxygen radical test-FORT), should be used in general clinical practice to set drug or nutritional therapies able to counteract OS in CKD patients [58]. According to the previous mentioned causes already discussed, redox state balance should be an important tool in the prevention of CV morbidity in the general population and, above all, in CKD patients [59].

### 3.5. Insulin Resistance

IR is typically defined as the inability of exogenous or endogenous insulin to increase glucose uptake and its utilization by target tissues, including skeletal muscles, the liver, and adipose tissue [60]. This condition results in hyperglycemia and compensatory hyperinsulinemia [61]. The alterations of insulin signaling pathways lead to the development of metabolic disorders, including diabetes mellitus, impaired glucose tolerance, obesity, dyslipidemia, and chronic low-grade inflammation. Nevertheless, these pathological conditions are also characterized by CV comorbidities and renal dysfunction, including arterial hypertension, coronary artery disease, atherosclerosis, and CKD. For this reason, they are better named cardiorenal metabolic syndrome, namely, pathological conditions that predispose to ED triggering [62]. In optimal conditions of insulin sensitivity, insulin, by binding with its cell surface receptor and the consequent activation of the phosphoinositide-3 kinase (PI3K)/akt signaling pathway, is capable of exerting both metabolic and vascular actions. The first, through an increased translocation of the glucose transporters type 4 (GLUT4), are able to reduce the blood glucose levels through increased glucose uptake by the adipose tissue and the skeletal muscle. The second action, instead, leads to an increase in NO production via eNOS enzyme activation. In fact, at the vascular endothelium level, the NO is able to exert a vasodilation response through capillary recruitment and increased blood flow, which contribute to the glucose uptake facilitating insulin action at the level of its organs’ target [63].

In CKD patients, chronic low-grade inflammation, metabolic acidosis, anemia, physical inactivity, vitamin D deficiency, and hormonal imbalance contribute to the onset of IR [64]. All of these CKD-associated comorbidities are able to cause IR by suppressing the insulin-receptor-mediated PI3K signaling pathway [65]. The lower GLUT4 translocation and the lower activation of the eNOS enzyme provoke both a reduced uptake of glucose and a reduced vasodilator response, thus developing into IR and ED. Moreover, in CKD patients, compensatory hyperinsulinemia creates an imbalance between prohypertensive and antihypertension vascular actions. In fact, the compensatory hyperinsulinemia, through the insulin-receptor-mediated mitogen-activated protein kinase (MAPK) signaling pathway (which is the least impaired pathway in the IR setting), leads to the production of ET-1, which, in turn, exerts an important vasocontraction action at the vascular endothelium level [66,67]. Moreover, the compensatory hyperinsulinemia induces the activation of the sympathetic nervous system, sodium reabsorption, cation pump activation, and vascular smooth muscle cell (VSMC) hypertrophy, which altogether cause a blood pressure increase, a phenomenon underlying ED [68].

The gut dysbiosis of CKD patients involves increased inflammation and epithelial barrier impairment, leading to a systemic translocation of gut-derived uremic toxins, which exert harmful effects via amplification of glucotoxicity, lipotoxicity, and systemic inflammation [69,70]. In particular, the glucotoxicity is involved in the increase in the ROS and hexosamine biosynthetic pathway (HSP) activity and the subsequent formation and buildup of AGEs [71]. The lipotoxicity, instead, is implicated in the OS increasing, the overactivation of proinflammatory signaling pathways, and the production of long-chain saturated fatty acids, called ceramides [72], which are potential biomarkers for coronary atherosclerosis [73]. Finally, systemic inflammation is characterized by an increase in proinflammatory factors [74]. All of the by-products of glucotoxicity, lipotoxicity, and systemic inflammation are capable of activating a variety of serine/threonine kinases, including I-kappa-β-kinase beta (IKKβ), NF-kβ, and activating protein-1 (AP-1), which directly or indirectly increase serine phosphorylation of IRS-1, resulting in a decreased PI3K/akt signaling pathway and, thus, in IR and ED [67].

In addition, in CKD patients, glucotoxicity, lipotoxicity, and systemic inflammation are capable of inducing ED through different mechanisms that do not involve the insulin receptor. In particular, regarding glucotoxicity, the AGEs are able to modify the ECM proteins, like collagen and laminin, leading to decreased vessel elasticity and increased macrophage infiltration. Infiltrated macrophages become foam cells that amplify vascular inflammation and promote atherosclerosis [5]. The protracted increase in glucose uptake by the glomerular and the proximal tubular cells leads to enhanced glucose flux through HSP, resulting in the formation of uridine diphosphate N-acetylglucosamine, which drives the O-GlcNAcylation of thousands of intracellular proteins, such as eNOS, causing the impairment of eNOS activity [75]. Moreover, the increased O-GlcNAcylation intensifies the OS, the apoptosis, and the activation of proinflammatory and profibrotic pathways through an increased expression of TGF-β, a relevant factor in ED pathogenesis [76,77]. Concerning the lipotoxicity, elevated levels of free fatty acids (FFAs) are able to stimulate NADPH oxidase, produce ROS, activate the NF-κB proinflammatory signaling pathway, inhibit eNOS activity, and increase endothelial cell proliferation. All of these mechanisms, induced by FFAs, affect the vascular wall through multiple events, including ED [78]. Finally, systemic inflammation, through the activation of NF-κB and the production of the proinflammatory cytokines, stimulates the expression of adhesion molecules, such as ICAM, VCAM, and E-selectin, which contribute to ED [79]. Likewise, high levels of CRP directly contribute to the pathogenesis of atherosclerosis and ED through reduced eNOS expression, the upregulation of the AT1 receptor in the endothelium [80], and the increase in the adhesion molecules’ expression [81].

### 3.6. Alteration of Calcium Phosphorus Metabolism

Calcium–phosphorus metabolism alterations cause mineral bone disorder (MBD), a typical comorbidity of CKD advanced stages. CKD–MBD can contribute to the onset of ED [12], and it is characterized by phosphorus retention, which, at high concentrations, leads to vascular calcifications and increases vascular stiffness. The enhanced vascular stiffness, in turn, is related to an increased CV risk in nephropathic patients [82]. In fact, uremic patients affected by CKD–MBD have a higher risk of developing vascular complications, such as the formation of atherosclerotic plaque, myocardial infarction, and post-angioplasty dissection [83]. An in vitro study evaluated the effects of inorganic phosphorus on vascular calcifications, highlighting how high concentrations of phosphorus, typical of uremic patients, induce ectopic calcifications, i.e., an anomalous mineralization of the soft tissues [84].

The calcification of blood vessels may involve either the media or the intima layers. Uremic patients often exhibit calcifications in the media layer. Specifically, the presence of vascular calcifications is linked to unfavorable clinical results, such as the eventual onset of myocardial ischemia and heart failure, induced by a reduced arterial elasticity, a heightened arterial rigidity, and an accelerated pulse wave velocity (PWV) [82,85]. Several studies have demonstrated that when the calcium/phosphorus product is increased, an enhancement of CV morbidity and mortality in dialysis patients is observed [86]. A direct correlation between Ca-P product value and the severity of the aortic insufficiency in dialysis patients has also been observed [82,87].

Furthermore, high concentrations of phosphorus can result in the conversion of VSMCs to an osteoblast-like phenotype, although the exact mechanism by which this happens is still unknown [88]. It has been hypothesized that this occurs following the increase in the expression of osteo-chondrogenic proteins and osteogenic genes. This increased expression occurs in the presence of high concentrations of phosphorus and calcium, which activate an intra-cellular signaling cascade [89]. This phenomenon is probably ascribable to the same mesenchymal origin between smooth muscle cells and osteoblast-like cells, which leads to the phenotypic transformation of the former. In fact, the development of vascular calcifications is intricate. It involves more than a mere deposition of calcium and phosphate. The VSMCs’ transformation into an osteoblast-like phenotype promotes matrix formation and attracts local factors that play an important role in the mineralization process. This is a dynamic process wherein VSMCs undergo apoptosis, which in turn causes the formation of microvesicles responsible for calcifications. [90]. Elevated phosphate levels enhance the activity of sodium-dependent cotransporters PiT-1 and PiT-2. This, in turn, induces the upregulation of the genes associated with matrix mineralization [91].

It was suggested that heightened intracellular phosphate levels might directly prompt the VSMCs’ transformation into calcifying cells by activating genes linked to osteoblastic functions. In fact, elevated phosphate levels play a significant role in increasing both the number and the activity of osteoclasts, thereby contributing significantly to increasing the bone resorption in CKD [92].

In addition, in the presence of pathological concentrations of inorganic phosphate, the transdifferentiation of VSMCs into calcifying cells is under the control of several non-coding RNAs, such as miR-223. Taibi et al. found that inorganic phosphate increased the levels of miR-223 in a vascular calcification in vitro model, and they also confirmed an increase in miR-223 in an in vivo model of calcified aortas of CKD mice. The same findings were detected in CKD patients [93].

Moreover, hyperphosphatemia seems to be related to the increase in the fibroblast growth factor 23 (FGF23) levels and to the decrease in its co-receptor expression, called Klotho, which are ED-inducing factors. FGF23 is a hormone synthesized by bone cells, such as osteoblasts and osteocytes, in response to elevated phosphate levels [94]. Increased circulating FGF23 levels contribute to phosphate-wasting disorders and robustly inhibit the expression of renal 1α-hydroxylase. This, in turn, diminishes the synthesis of vitamin D’s active form (1α,25-dihydroxyvitamin-D3) in the renal proximal tubules. Vitamin D deficiency, as well as the alteration of the calcium–phosphorus balance, are considered potential ED risk factors. In fact, vitamin D is involved in the regulation of NO synthesis by mediating eNOS activity [95].

Furthermore, several studies have demonstrated how vitamin D deficiency is associated with chronic low-grade inflammation in chronic non-degenerative communicable disease, thus synergically amplifying ED [96,97,98].

Finally, hyperphosphatemia can induce an increase in OS and an alteration in NO’s bioavailability [12]. In fact, in nephropathic patients with hyperphosphatemia, a reduction in the activity of iNOS and an increase in the activity of protein kinase C are observed, which are responsible for ROS production and the inhibition of iNOS expression [99].

### 3.7. Early Aging

It is well-known that CKD is characterized by traditional and non-traditional CV risk factors. Among the latter, early aging plays a key role [100]. In fact, aging is one of the main risk factors for the onset of numerous pathologies, especially chronic degenerative non-communicable ones and their related comorbidities.

Among the factors that influence early aging, there are OS and systemic inflammation. These conditions are exacerbated during CKD and cause earlier aging of nephropathic patients compared to the general population.

The speed with which aging occurs depends on numerous factors, which are both genetic and environmental [101]. Among the genetic factors, the role of extracellular vesicles, which are physiologically responsible for cellular communication, has recently been evaluated. During CKD, due to IS accumulation, there is an increase in the levels of endothelial-derived extracellular vesicles, which transport microRNA (miR) [102]. The overexpression of miRs in CKD patients induces immune disorders, chronic inflammation, and ED [103].

Among the environmental factors, chronic pain has also recently been described. In fact, it appears to significantly influence the telomere length [104]. CKD patients exhibit early aging due to stress signals that induce cellular apoptosis. Physiologically, cells have an anticoagulant and non-adherent surface; however, during CKD, the alteration of molecules expressed on the surface of endothelial cells can be impaired, thus increasing cell adhesion [105]. This phenomenon determines a hyper-coagulative state characterized by thrombosis and inflammation [106].

## 4. Methods for Diagnosing Endothelial Dysfunction

**Figure 2:**
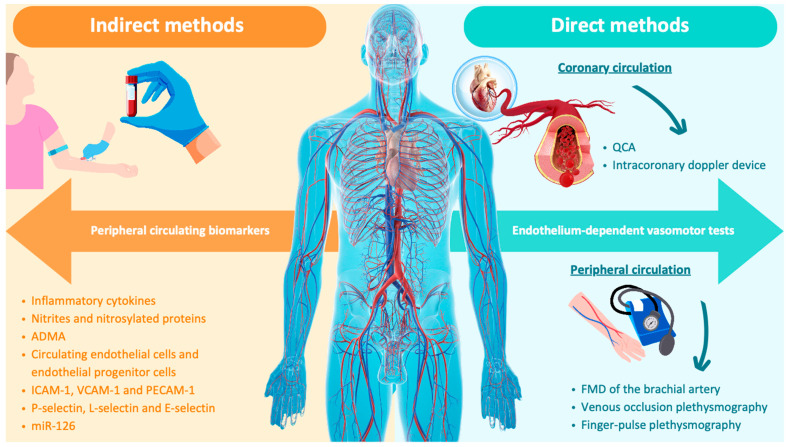
Methods for diagnosing endothelial dysfunction. Studying endothelial function is based on two main methods: indirect and direct. The first one consists of measuring the concentration of peripheral circulating biomarkers. The second method is based on endothelium-dependent vasomotor tests in order to assess coronary circulation and/or peripheral circulation. Abbreviations: ADMA, asymmetric dimethylarginine; FMD, flow-mediated dilatation; ICAM-1, intracellular adhesion molecule-1; miR-126, microRNA-126; PECAM-1, platelet endothelial cell adhesion molecule-1; QCA, quantitative coronary angiography; VCAM-1, vascular cellular adhesion molecule-1.

The study of endothelial function in vivo is based on two methods (Figure 2).

The first one is an indirect method, while the second is a direct method. In particular, the first is based on the measurement of specific ED biomarkers, whereas the second is based on the response of the endothelium to vasomotor tests.

In the detail, the first consists of the measurement of the concentration of peripheral circulating markers using indirect information regarding the state of the endothelium. These markers include direct products of endothelial cells, such as (i) inflammatory cytokines, (ii) nitrites and nitrosylated proteins, which in part reflect endothelial generation of NO [107], (iii) ADMA, whose levels are elevated in CKD patients and in subjects with high CV risk and preclinical atherosclerosis disease [108,109], (iv) circulating endothelial cells and endothelial progenitor cells, detected through flow cytometry, which are considered markers of endothelial damage and repair [110,111], (v) adhesion molecules, namely ICAM-1, VCAM-1, and platelet endothelial cell adhesion molecule-1 (PECAM-1), which are able to predict the presence of adverse CV events [81], (vi) selectins, like P-selectin, L-selectin, and E-selectin, and (vii) miR-126, whose lower serum levels in CKD patients have been associated with higher levels of several ED biomarkers, namely the syndecan-1 and the free-indoxyl sulfate [112].

Due to their difficult to dose and their cost, these circulating markers are currently used for research purposes and, furthermore, only a small quantity of them (around 20%) are released into the bloodstream [113].

The second method, on the other hand, is able to provide direct information on the functional capacity of the endothelium. This method is based on endothelium-dependent vasomotor tests that allow for evaluating the response of endothelial cells through the stimulation by specific agonists or antagonists [114]. These molecules are infused in the coronary arteries in order to study the endothelial function of coronary circulation or in the brachial artery in order to study the endothelial function of peripheral circulation without any changes in the systemic hemodynamic [115]. Endothelial agonists (such as acetylcholine, bradykinin, substance P, serotonin, and adenosine) possess specific endothelium receptors and are capable of stimulating the production of NO [116], while antagonist (such as NG-Monomethyl-L-arginine- L-NMMA), as with those for the NOS enzyme, can block NO production [117]. The endothelial function of the coronary circulation can be investigated through either quantitative coronary angiography (QCA) or using an intracoronary Doppler device. The QCA evaluates the vascular diameter change of the anterior interventricular artery, while the intracoronary Doppler device is used to study the coronary microcirculation blood flow and the coronary vascular resistance [118].

The endothelium is considered physiologically healthy when agonist molecules cause NO-mediated vasodilation. On the contrary, the presence of ED is detected by the absence of vasodilation or the paradoxical vasoconstriction. The degree of vasodilation is considered a measure of endothelial function, so the greater the vasodilation, the higher the endothelial function [119].

The study of coronary endothelial function, thanks to its sensitivity, accuracy, repeatability, and reproducibility, is considered the gold standard technique to evaluate if coronary ED is present [120]. However, due to its complexity and its invasiveness, this procedure is not used as a screening test in the low-risk population. The endothelial function of the peripheral circulation can be studied by measuring flow-mediated dilatation (FMD) of the brachial artery by using high-resolution ultrasound [121]. This technique consists of measuring the brachial artery diameter before and after an increase in shear stress because of local endothelial NO release [122,123]. The latter is induced by reactive hyperemia provoked using a sphygmomanometer cuff placed proximal to the brachial artery and inflated up to 200 mmHg for a period of 5 minutes. FMD investigates the endothelial vasodilator properties at the peripheral site, which reflect the coronary endothelial function and therefore the vascular NO’s bioavailability [124]. In fact, several studies have shown a correlation between the brachial artery FMD and the carotid intima-media thickness [125,126]. Peripheral circulation can also be investigated through venous occlusion plethysmography or finger-pulse plethysmography [120,127]. The first method uses the plethysmograph to measure forearm volume changes, which depend on the arterial blood flow. This technique uses two cuffs, inflated at the upper arm and wrist, which block venous drainage and simultaneously exploit an intrabrachial infusion of endothelium-dependent vasodilators. Venous occlusion plethysmography has been widely used in the past, but nowadays it has been abandoned due to its invasiveness and its poor reproducibility [128]. The second method uses a fingertip plethysmograph through the pulse amplitude tonometry (PAT) to quantify the arterial pulse volume at rest and during conditions of hyperemia due to the increase in the shear stress provoked by 5 minutes of insufflation of a sphygmomanometer cuff at the forearm level, as seen in FMD [129].

The reactive hyperemia index (RHI) is given by the ratio of the PAT value at rest and the PAT value after reactive hyperemia [130]. The study of peripheral endothelial function through FMD, at the forearm level, is the most used method in clinical practice, thanks to its non-invasiveness, cheapness, and safety [131]. Moreover, it is worth considering that even if the coronary district appears to be more susceptible to developing ED than the peripheral district, ED is a systemic disorder, and there seems to be a correlation between the two districts. In fact, peripheral ED is always associated with coronary ED [6]. However, the FMD test has numerous limitations, as changes in the brachial artery caliber are reduced, and numerous technical measures are required to give this method better reproducibility [132]. In order to increase the FMD’s reproducibility, additional precautions by the operator should be required. Some of them should be (i) standardizing the assessment time (preferably in the morning), (ii) fasting for at least 8 hours before the endothelial function study, (iii) no smoking and no use vasoactive drugs the morning of the measurements, and (iv) awareness that endothelial function can also be influenced by hormonal factors, such as the menstrual cycle, mental stress, and sleep deprivation [133,134]. In addition, not all cases of coronary ED are associated with peripheral ED. For all of these reasons, no method able to diagnose ED can be considered a surrogate for another one. Therefore, nowadays, no definitive diagnostic conclusion can be drawn from a single technique assessing endothelial function [135].

## 5. Innovative and Traditional Treatments

ED has an important role in the kidney’s microvascular hemodynamics, tubulo-glomerular feedback, and natriuresis. Therefore, it is involved not only in the development but also the exacerbation of albuminuria in CKD progression [136]. In a previous study, Clausen et al. demonstrated a significant and direct association between impaired FMD and elevated urinary albumin excretion [137]. Several clinical trials are ongoing to evaluate the effect of selected pharmacological interventions on endothelial function, specifically in CKD patients. Direct and indirect endothelial protective effects of antihypertensive drugs (i.e., calcium channel blockers, angiotensin-converting enzyme ACE inhibitors, angiotensin II receptor blockers, and ultra-selective 1-blockers, such as nebivolol) [5], lipid-lowering (i.e., statin) and hypoglycemic drugs, commonly used in CKD patients, have been widely documented [138].

Innovative approaches to counteract ED can also include nutritional therapy and adapted physical activity programs [12]. Unlike pharmacological treatments, which require adjustment of the dosage based on renal and hepatic function, an adjuvant therapy based on NBCs is free from side effects if it respects the recommended intakes, despite the renal and hepatic function [139,140]. Both innovative and traditional treatments are reported in Table 1.

### 5.1. SGLT-2 Inhibitors

Recently, a novel class of glucose-lowering drugs, called sodium glucose co-transporter 2 inhibitors (SGLT-2is), have been associated with an improvement in CV and renal outcomes, irrespective of the presence of diabetes mellitus [141].

The primary nephroprotective mechanism of SGLT-2is is the increase in distal sodium delivery and the inhibition of tubule-glomerular feedback, resulting in arteriolar afferent vasoconstriction and reduction of intraglomerular pressure, with a consequent reduction in albuminuria [142].

Natriuresis and the subsequent contraction of plasma volume lead to a decrease in blood pressure and arterial stiffness, with a significant ED reduction [143].

SGLT-2is exerts protective effects on the endothelial cells via several pathways: (i) the inhibition of OS, thus counteracting ROS production; (ii) the prevention of the inflammatory reaction, thus re-establishing the correct NO bioavailability; (iii) the mitigation of mitochondrial injury; and (iv) the modulation of angiogenesis and cellular senescence.

SGLT-2is’s anti-inflammatory effects include a reduction in cytokine secretion, the downregulation of ICAM-1 and VCAM-1, and the prevention of the adhesion of the monocytes at the endothelium [144].

In preclinical studies, SGLT-2is was demonstrated to prevent ED. In particular, empagliflozin decreased aortic stiffness by promoting glycosuria in a mouse model of T2DM [145], and, in another model, it mitigated the endothelial senescence induced by high glucose levels, thus inhibiting the local renin–angiotensin–aldosterone system [146]. Dapagliflozin reduced arterial stiffness in a diabetic model and improved diastolic function in non-diabetic mice [147,148]. Moreover, SGLT2is ameliorated the inflammatory phenotype and glucotoxicity by acting on AGE/RAGE signaling in diabetic models [149].

Indeed, several ex vivo and in vivo studies support a SGLT-2is class effect on the regulation of the endothelial function [144]. In fact, SGLT-2is also seems to induce a positive impact on the non-invasive vascular function tests, such as FMD and PWV.

A meta-analysis including 26 clinical studies assessed the effects of different types of antidiabetic drugs, including dipeptidyl peptidase-4 (DPP-4) inhibitors, GLP-1-Ras, and SGLT2is, on FMD. The authors concluded that only SGLT2is significantly enhanced the FMD [150]. However, further studies are necessary in order to confirm these interesting data.

### 5.2. Mineralocorticoid Receptor Blockers

Aldosterone increases OS in the vascular wall, thus acting on the mineralocorticoid receptor (MR) and contributing to ED development [151]. Commonly used steroidal MR antagonists (MRAs), like spironolactone and eplerenone, are effective in reducing ED [152], but in patients with an advanced CKD stage, their use is limited because of the risk of severe hyperkaliemia and faster progression until ESRD.

Finerenone, a novel highly selective non-steroidal MRA, is able to counteract both ED and albuminuria. It shows a similar potency compared to spironolactone and a greater selectivity compared to eplerenone towards MR [153]. This drug is also characterized by a significantly smaller increase in kaliemia and a lower decrease in the estimated glomerular filtration rate (eGFR) [154]. For these reasons, it can be used in patients with mild to moderate CKD. Finerenone acts by determining an upregulation of the Mn–superoxide dismutase, with a consequent decrease in superoxide anion levels and an enhancement of NO’s bioavailability [155].

### 5.3. Nutritional Treatments and Adapted Physical Activity

It is well-known and widely documented that for an ideal clinical management of CKD patients under conservative therapy, nutritional therapy, based on reduced protein intake, which varies between 0.6 and 0.8 g/kg of observed or ideal body weight (b.w.)/day, represents an important tool to be combined with pharmacological treatment. This complete approach is successful for the management of CKD-related complications and for preventing its progression to ESRD [156]. In fact, a low-protein-diet (LPD), based on 0.6 g/kg of observed or ideal b.w./day of protein intake, is able to reduce uremic toxin accumulation, counteract metabolic acidosis, restore calcium–phosphorus metabolism and the gut microbiota eubiosis, increase insulin sensitivity, and decrease OS and arterial blood pressure values [157]. It has been widely discussed how all of these factors are involved in ED pathogenesis in CKD patients. However, in this patient population, in order to prevent malnutrition, a normo-mildly hypercaloric diet is strongly recommended [158]. Extra virgin olive oil (EVOO), a lipid food that constitutes a pillar of the MD, plays a crucial role in the clinical management of CKD patients and, in particular, in counteracting PEW syndrome, a comorbidity typical of CKD patients [159]. EVOO’s beneficial effects are attributable to its chemical composition, including fatty acids (98–99%) and other bioactive compounds (1–2%), such as minor polar compounds (MPCs), especially of the secoiridoid and phenolic variety. Among these, oleocanthal (OLE), oleacin, hydroxytyrosol (HT), tyrosol, and HT glycole are of particular importance [160]. Several studies have demonstrated how daily consumption of EVOO exerts beneficial effects on human health, including, in particular, in relation to CV and metabolic diseases [161]. For this reason, the MPCs’ health value contained in EVOO was reinforced by the European Food Safety Authority (EFSA) in 2011. The EFSA, through European Commission Regulation no. 432/2012, granted permission to put a health claim on the label concerning EVOO’s polyphenols’ efficacy in counteracting LDL oxidation [162]. The latter action refers to the initial event that leads to atherosclerotic plaque formation through alterations in the eNOS signaling pathway [163]. EVOO’s positive effect is achieved by daily consumption of 20 g containing at least 5 mg of HT and derivatives (e.g., oleuropein complex and tyrosol) (5 mg/day per 20 g of EVOO) [162]. However, we believe that the health effects induced by the intake of EVOO, which complies with the EFSA’s health claim, may refer to real CV protection. In fact, in the study conducted by Marrone et al., intake of 40 mL/day of raw EVOO for 9 weeks, containing a very high content (>900 ppm) of MPCs, was also able to exert a cardioprotective action in CKD patients. At the end of the study, the authors highlighted (i) a significant increase in high-density lipoprotein (HDL) cholesterol; (ii) a significant reduction in values related to OS and anti-inflammatory parameters, monitored in relation to FORT, CRP, TNF-α, and IL-6; (iii) a significant decrease in atherogenic indices, calculated through total cholesterol/HDL-cholesterol, LDL-cholesterol/HDL-cholesterol, and log triglycerides/HDL-cholesterol); (iv) a significant reduction of other inflammatory parameters, like the platelet-to-lymphocyte ratio, the neutrophil-to-lymphocyte ratio, the lymphocyte-to-monocyte ratio, and the lymphocyte count; and (v) a significant reduction in carotid intima-media thickness [164].

In addition to HT, another important EVOO MPC is OLE, which is characterized by anti-inflammatory action. In fact, this compound is homologous to ibuprofen, a non-steroidal anti-inflammatory drug (NSAID) that is able to inhibit COX enzymes [160,165]. Several studies demonstrate that a chronic low dose of ibuprofen and other COX inhibitors (like aspirin) exerts anti-neoplastic and anti-thrombotic effects [166,167,168]. Therefore, it may be speculated that a low dose of OLE may counteract the platelets’ aggregation and, consequently, ED. An interesting study conducted by Beauchamp et al. highlighted that OLE is not only able to inhibit COX enzymes in the same manner as ibuprofen but also in a more effective manner. In fact, at equimolar concentrations, the inhibiting action of OLE is significantly higher compared to ibuprofen [169].

All of the scientific studies regarding the ideal nutritional treatment for metabolic patients agree that dietary patterns that include more plant-derived foods and less saturated fats (mainly present in animal products) are able to improve high blood pressure values, high serum total cholesterol, glucose metabolism, gut microbial dysbiosis, and chronic low-grade inflammation [170].

In CKD patients, the first researcher who talked about nutritional therapy characterized by the consumption of plant-based sources was Professor Kalantar-Zadeh. He defined the plant-dominant (PLADO) LPD, a diet consisting of >50% plant-based sources, sodium intake <4g/day (<3 g/day if CKD is accompanied by edema or hypertension), fiber intake >25 g/day, and energy intake between 30 and 35 kcal of observed or ideal b.w./day.

In this review, we focus only on reporting the beneficial CV outcomes induced by PLADO LPD. In particular, this nutritional therapy exhibits the following cardioprotective effects: (i) anti-inflammatory and antioxidant effects due to a higher intake of anti-inflammatory and antioxidant plant-based food rich in polyphenols; (ii) better control of metabolic pathways producing AGEs due to an higher dietary fiber intake; (iii) minor production of gut-derived uremic toxins (such as IS and pCS) due to a reduction in nitrogenous compounds contained in plant-based foods; and iv) reduction of metabolites derived from the gut bacteria (such as trimethylamine-TMA and TMAO) [171].

Because the PLADO LPD is mainly based on the legume consumption as a plant-based protein source, it is worth emphasizing the cardiometabolic effects of this food category. The actions exerted by legumes on ED can be summarized by the improvement of (i) lipid metabolism, monitored by total cholesterol, LDL-cholesterol, triglycerides, and HDL-cholesterol; (ii) inflammation, detected by a reduction in CRP levels; and (iii) vasodilation, monitored by an increase in eNOS and NO bioavailability and a reduction in TNF-α and Ang II gene expression [172].

However, in CKD patients, in order to enhance the beneficial effects for ED exerted by the PLADO LPD, an adapted physical activity (APA) program is strongly recommended. Growing evidence of the beneficial effects of exercise training for ED in CKD patients is present in the literature [173,174]. Aerobic exercise favorably impacts the levels of ET-1, NO, and other vasoactive substances [175,176,177], thus normalizing plasma ET-1 levels [178]. Common to ED is excessive OS, which plays an important role in those processes underlying vascular changes. Moderate to vigorous aerobic exercise improves the redox state and, consequently, NO’s bioavailability, thus ameliorating microvascular endothelial function, maintaining the artery vessels’ function, preventing the progression of vascular diseases, facilitating substrate delivery for NO production, reversing impairment to the L-arginine transport system [179], and reducing CV morbidity and mortality in CKD [174]. It was also shown that resistance training (RT) evokes NO release, thus reducing ADMA and improving redox and inflammatory profiles [180].

In conclusion, as suggested in the literature, APA may represent an established stimulus, an attractive, well-recommended, non-pharmacological strategy, and a new tool to counteract ED in CKD.

### 5.4. Use of Ketoanalogues

During amino acid degradation, transamination, catalyzed by the aminotransferase enzymes, involves the removal of the amino group (NH_2_) bound to the α-carbon from the rest of the amino acid carbon skeleton and its replacement by a keto or hydroxyl group. Further steps lead to ammonia production, which is rapidly converted into urea and eliminated with the urine [181]. In CKD patients, increased urea concentration and its consequently augmented flux into the colon lead to gut microbiota dysbiosis.

The increase in proteolytic bacteria at the expense of the saccharolytic ones is able to produce gut-derived uremic toxins, resulting in an impairment of the epithelial tight-junctions and in an intestinal permeability enhancement that induces the bacterial translocation in blood circulation (a phenomenon also called “bacterial endotoxemia”) [182]. This crosstalk between uremia and gut microbiota dysbiosis seems to explain the chronic low-grade inflammation in CKD patients, thus resulting in faster CKD progression [183].

In patients with CKD stages 4–5, according to the Kidney Disease Improving Global Outcomes (KIDGO) guidelines, a very low-protein diet (VLPD) providing 0.3–0.4 g of protein/kg of observed or ideal b.w./day is used to delay the necessity of renal replacement therapy.

In order to reduce the uremic toxins’ accumulation and maintain good nutritional status, the VLPD must necessarily be supplemented with alpha ketoacid or alpha hydroxyacid analogs of an essential amino acid (EAA) (ketoanalogues-KAs), with approximately one tablet per 5 kg of observed or ideal b.w./day [184].

Another innovative therapeutic option for the clinical management of ESRD patients is incremental dialysis, namely a renal replacement therapy scheme, characterized by a single-weekly dialysis treatment combined with a high-protein diet on the dialysis day (1.2 g of protein/kg of observed or ideal b.w./day) and a VLDP supplemented with KAs on the non-dialysis days. This therapeutic approach is ideal for hemodialysis patients with residual renal function [185].

In contrast with its mandatory use in the VLDP, the supplementation of KAs in the LPD is optional, as recommended by the authorities’ consensus [186].

Because KAs lack the amino group linked to the α-carbon of an amino acid, they can be converted to their respective amino acid without providing an additional nitrogen load. Once they are aminated by the amino groups of the amino acids, KA supplements provide a nutritional source of EAA, resulting in a reduction in urea synthesis and other nitrogen-containing potential toxins. The amino acid carbon skeletons can be degraded without a net production of nitrogenous waste products or may be used to reform amino acids.

The decreased amino acids’ degradation and the recycling of the amino groups, together with the reduced urea synthesis, are successful in (i) preventing malnutrition; (ii) improving insulin sensitivity, calcium–phosphate metabolism, the lipid profile, and quality of life; (iii) slowing the progression of CKD; and (iv) decreasing uremic toxins [186].

Unfortunately, few studies to date have investigated the positive effects of LPD or VLPD supplemented with KAs for ED in CKD patients. In the study conducted by Chang et al., the administration of the LPD combined with KA supplementation (6 tab/day) for 6 months in CKD patients (stage 3b–4) was able to decrease IS and pCS levels, thus leading to an increase in FMD of the brachial artery, which reflects the amount of NO production. At the end of the study, the authors highlighted a significant increase in eGFR due to the reduced endothelial damage, which was probably related to the IS and pCS decrease [187]. In another study conducted on 111 CKD patients (stage 3–4) with obesity (body mass index (BMI) ≥ 30 kg/m^2^ and waist/hip ratio > 0.85) for 36 months, Teplan et al. evaluated the effects of the LPD supplemented with KAs (at the dose of 100 mg/kg b.w./day) on ED plasma markers, including ADMA. As previously discussed, elevated ADMA levels in CKD patients have been detected, and they seem to be implicated in ED pathogenesis. However, in CKD patients, elevated ADMA levels may depend not only on CKD itself but may also be influenced by the presence of other CKD-related comorbidities, such as obesity, which is considered one of the main CKD risk factors. The principal ED markers, assessed by the authors, in addition to ADMA, were adiponectin and pentosidine. High levels of adiponectin protect the vascular endothelial function through anti-atherosclerotic and anti-inflammatory actions, while pentosidine is an AGE that contributes to CV disease. At the end of the above-mentioned study, the authors pointed out how, in CKD patients with obesity, long-term LPD administration, supplemented with KAs, led to a significant reduction in ADMA and pentosidine levels and a significant increase in adiponectin levels compared to the control group. Moreover, in the study group, the combined approach was able to significantly reduce BMI and significantly delay the decline of renal function. The ameliorations in ADMA, pentosidine, and adiponectin levels were closely linked to BMI. In fact, the relationship of these three parameters was mainly associated with improved BMI and then with GFR. Furthermore, in the study group, the decrease in body fat was accompanied by an improvement in lipid metabolism through a significant reduction in total cholesterol, LDL-cholesterol, and triglycerides values and an improvement in glucose metabolism, detected by a significant reduction in glycated hemoglobin levels. Finally, the administration of the LPD supplemented with KAs was also associated with a significant reduction in systolic and diastolic blood pressure and in proteinuria values. The authors hypothesized that by decreasing the BMI and visceral fat, cells are not able to synthesize ADMA, while a better glycemic metabolism can improve DDAH activity and, thus, further reduce ADMA levels [188].

It is worth concluding that an LPD supplemented with KAs seems to significantly delay the progression of kidney damage and exert beneficial effects on ED and the accumulation of protein-bound uremic toxins in CKD patients, affected or not by obesity.

**Table 1:** Innovative and traditional treatments for endothelial dysfunction.

Type of Treatment	Mechanisms of Action	Beneficial Effects on Endothelial Function	Bibliography
SGLT-2 inhibitors	↑ distal sodium delivery ↓ tubule – glomerular feedback ↓ ROS production downregulation of ICAM-1 and VCAM-1 ↓ mitochondrial injury modulation of angiogenesis and cellular senescence ↓ renin–angiotensin–aldosterone system activity	↓plasma volume and blood pressure ↓arterial stiffness ↓ inflammation and oxidative stress ↑ NO bioavailability	[143,144]
Mineralcorticoid receptor blockers	↓ aldosterone action upregulation of the superoxide dismutase	↓ superoxide anion levels ↑ NO bioavailability	[151,152,155]
Nutritional treatments:			
A) Extra virgin olive oil	↓ LDL oxidation ↓ ROS production ↓ IL-6, TNF-α ↓ COX enzymes activity ↓ eNOS activity	↓ atherosclerotic plaque formation ↓ inflammation and oxidative stress ↑ NO bioavailability	[160,161,162,163]
B) PLADO diet	↑ ROS production ↑AGEs production ↑gut-derived uremic toxins ↑ Ang II gene expression	↑ inflammation and oxidative stress improvement in lipid metabolism ↑ NO bioavailability	[171,172]
C) Use of ketoanalogues	↓ IS and pCS levels ↓ ADMA ↑ adiponectin ↓ pentosidine ↓ glycated hemoglobin levels ↓ uremic toxins	↓ inflammation and oxidative stress improvement in lipid and glucose metabolism ↓ systolic and diastolic blood pressure	[187,188]
Adapted physical activity	normalizing ET-1 levels ↓ ADMA	↑ NO bioavailability ↓ inflammation and oxidative stress	[175,176,177,178,180]

*Abbreviations:* SGLT, sodium glucose co-transporter 2 inhibitors; ROS, reactive oxygen species; NO, nitric oxide; ICAM-1, intracellular adhesion molecule-1; VCAM-1, vascular cellular adhesion molecule-1; LDL, high-density lipoproteins; IL, interleukin; TNF, tumor necrosis factor; COX, cyclooxygsenase; PLADO, plant-dominant; Ang, angiotensin; ET, endothelin; ADMA, asymmetric dimethylarginine; IS, Indoxyl sulfate; pCS, p-cresyl sulfate; ↑ increase; ↓ decrease.

## 6. Conclusions

CV diseases are one of the most frequent comorbidities in CKD patients due to several CV risk factors underlying the pathogenesis of CKD itself. ED represents the primum movens of the CV disease, and, in nephropathic patients, several mechanisms of action contribute to ED onset. These pathogenic mechanisms seem to compromise the production of a series of endothelium-derived relaxing factors responsible for the maintenance of vascular homeostasis. Although the study of the coronary endothelial function is considered the gold standard to assess if ED is present, no methods among those able to diagnose ED can be considered a surrogate for the other ones. Once ED has been identified, its treatment necessarily requires both a traditional pharmacological treatment but also an adjuvant non-pharmacological therapy. Concerning pharmacological therapy, in this review, we focused on describing the beneficial role that MRA and SGLT-2is exert on endothelial cells. In particular, the first are capable of reducing superoxide anion levels and increasing NO bioavailability, while the second are capable of reducing the blood pressure, arterial stiffness, and inflammation, thus restoring the correct NO bioavailability and counteracting ROS production. All of these pharmacological effects produce an improvement in the FMD and the PWV. In CKD patients, in order to set an appropriate pharmacological and adjuvant non-pharmacological therapy, OS monitoring through the FORT should also be used in general clinical practice.

Among innovative non-pharmacological therapies, in this review, we wanted to explain how EVOO rich in MPCs is able to exert CV protection in CKD patients. However, only EVOOs that comply with the EFSA health claim are able to protect the endothelium from LDL oxidation. In CKD patients, these beneficial effects can be enhanced through adherence to the PLADO LPD, whose main characteristic is the inclusion of at least 50% plant-based proteins. Several studies have highlighted how vegetable proteins are capable of improving lipid metabolism, inflammation, and vasodilation through an increase in eNOS activity and NO bioavailability and through a reduction in the TNF-α and Ang II gene expression. The triad can be completed through adherence to an AFA program, which several studies showed to be able to determine a balance between substances with vasodilating and vasoconstrictive action, improve NO bioavailability, ameliorate microvascular endothelial function, and reduce ADMA levels. These measures also become essential for the clinical management of patients at an advanced CKD stage. However, with this review, we also wanted to elucidate the role of KAs in ED. These latter may be taken optionally when combined with the LPD, while they must be taken when combined with the VLPD. Few studies to date have highlighted the beneficial effects induced by the LPD or the VLPD combined with KA supplementation for ED. However, it has been demonstrated that the LPD supplemented with KAs has been able to increase FMD of the brachial artery and DDAH activity, reduce ADMA and pentosidine levels, and increase adiponectin ones. In conclusion, we believe that it is very important evaluate the impact of the combination of all adjuvant treatments, as previously described, on ED in CKD patients through a randomized clinical trial conducted on a large sample size.

## Data Availability

Not applicable.
